# The Neural Basis of Mark Making: A Functional MRI Study of Drawing

**DOI:** 10.1371/journal.pone.0108628

**Published:** 2014-10-01

**Authors:** Ye Yuan, Steven Brown

**Affiliations:** Department of Psychology, Neuroscience & Behaviour, McMaster University, Hamilton, Ontario, Canada; University of Tokyo, Japan

## Abstract

Compared to most other forms of visually-guided motor activity, drawing is unique in that it “leaves a trail behind” in the form of the emanating image. We took advantage of an MRI-compatible drawing tablet in order to examine both the motor production and perceptual emanation of images. Subjects participated in a series of mark making tasks in which they were cued to draw geometric patterns on the tablet's surface. The critical comparison was between when visual feedback was displayed (image generation) versus when it was not (no image generation). This contrast revealed an occipito-parietal stream involved in motion-based perception of the emerging image, including areas V5/MT+, LO, V3A, and the posterior part of the intraparietal sulcus. Interestingly, when subjects passively viewed animations of visual patterns emerging on the projected surface, all of the sensorimotor network involved in drawing was strongly activated, with the exception of the primary motor cortex. These results argue that the origin of the human capacity to draw and write involves not only motor skills for tool use but also motor-sensory links between drawing movements and the visual images that emanate from them in real time.

## Introduction

Images refer to visual patterns created on surfaces, generally on flat surfaces (such as canvases or cave walls) but also on three-dimensional objects (such as human bodies or ceramic vases). This includes the products of both drawing and writing as well as a third category of images that Elkins [1] refers to as “notation”, including musical notation, mathematical formulas, and a host of other images that are categorized as neither pictures (drawing) nor words (writing).

From a motor-control perspective, drawing can be thought of as being similar to most other forms of visuo-manual activity, including the ones that neuroscientists typically study, such as reaching, grasping, object manipulation, pointing, and gesturing [2]. It involves visual guidance of hand movement towards a target though hand/eye coordination. Drawing also shows similarities with forms of tool use (e.g., joystick movement), as it invariably involves use of a drawing tool. Drawing, therefore, is similar to other forms of manual activity in that it is a dynamic sensorimotor process.

However, it differs from all these forms of motor activity in an important respect: *it leaves a trail behind*. In other words, an image emerges through the process of performing the motor activity. We will use the term “emanation” to refer to this emergence of an image as drawing progresses. Emanation applies to writing as much as drawing, since writing too is a form of image generation. Therefore, while pictures themselves are generally considered to be static objects – making them standard stimuli for studies of “neuroaesthetics” [3] – drawing itself is a dynamic process both in the sense that it requires visually-guided coordination of the eyes, hands and body, and more uniquely that it involves emanation of an image, in other words the intentional laying down of a trail on a surface as the movement occurs.

Many previous neuroimaging studies of drawing have had a strong limitation in that subjects’ perception of emanation during drawing was limited by a lack of visual feedback in the scanning situation. The major modalities for drawing in these studies included drawing in the air [4] and drawing on a pad sitting on the body or on a writing board using a drawing tool [5]–[8], [9] or a finger alone [10]. Several studies have had subjects draw covertly using mental imagery alone [5], [11], [12]. In some studies of overt production, the eyes were kept closed during the drawing task [7], [10]. In certain studies in which the eyes were open, no visual activations were reported [4]. Curiously enough, the very first imaging study of drawing [13] performed a tracing task using positron emission tomography (PET) in which subjects were indeed able to see their tracings via a back-projection system (see also [9], [14]). Studies of air drawing and imagined drawing, beyond having feedback limitations, provide no behavioral data on subject performance (whereas pad studies produce drawings).

More recently, a small number of drawing studies have used MRI-compatible drawing devices that are able to provide visual feedback to subjects during drawing, thus permitting the capacity to perform tracing tasks in the MRI scanner. These include the use of an MRI-compatible mouse [15]–[16] and drawing tablet [17]–[20]. The current study took advantage of the MRI-compatible drawing tablet devised by Tam et al. [19]. This tablet not only provides visual feedback to subjects but furnishes a means of recording all the drawing movements of the subject, permitting video reconstruction of drawing trajectories and thus behavioral performance during drawing. It also provides a means of manipulating visual feedback to the subject during drawing, for example the ability to eliminate visual feedback, as was done in the present study (see also Thaler & Goodale [20]).

Drawing can occur in three principal ways: from memory, as copying, or as tracing. Whereas writing is almost always done from memory, drawing is done equally commonly as copying (for example, drawing a sitter’s portrait) and as drawing from memory. In like form, most neuroimaging studies of drawing have had subjects generate images either from memory [6]–[8], [10], [14], [16], [21] or as a copying task [4], [5], [8], [9], [11], [14], [16], [18]. Tracing has been restricted to the few studies that have provided visual feedback to subjects in the scanner [13]–[16], [18].

The abovementioned neuroimaging literature for drawing has produced a reliable set of findings. This includes not only expected activations in the left primary motor cortex and right cerebellum for right-handed drawing but quite often activity in the posterior parietal cortex, including the cortex of the intraparietal sulcus (IPS). The IPS is involved in creating a transformation between retinotopic coordinates in visual space and egocentric motor coordinates in effector space, thereby supporting visual guidance of hand movement [22]. Such activity must be coordinated with eye movement as well, since eye position defines retinotopic position. Posterior parietal activations tend to either be either ipsilateral to the motor-cortex activations or bilateral. Other common activations have been found in the frontal eye fields (FEF), inferior frontal gyrus (IFG), and precuneus. Studies that have provided visual feedback to subjects have been the most informative since they have observed visual activations as well. For example, Ogawa and Inui [16] had subjects perform both copying and tracing of curved lines using an MRI-compatible mouse with visual feedback. While tracing gave no residual activations beyond copying, copying gave additional activations in V1, V2, IPS, IFG and pre-supplementary motor area (SMA), most likely reflecting the greater spatial demands of copying compared to tracing in recreating the visual properties of the drawn object.

The principal objective of the current study was to examine the neural basis of image generation and its emanative component through the performance of mark making tasks while taking advantage of the precision and flexibility conferred by using an MRI-compatible drawing tablet, not least the ability of subjects to see what they were drawing and for visual feedback to be manipulated. A critical comparison was between when visual feedback was displayed on the projected screen (image generation) versus when it was not (“blind drawing”, the situation of many previous imaging studies of drawing). This contrast should allow us to isolate brain areas important for emanation in drawing. In a perceptual control condition, we had subjects passively view an animation of an image emerging in time on the projected surface. This motion-perception task should likewise reveal brain areas important for emanation. Finally, we included the additional condition of copying in order to examine a drawing task that has a stronger spatial-processing demand than a task done from memory. We predicted that, unlike most previous studies of drawing, we would observe activations in parts of the brain involved in motion perception, eye movement, and hand/eye coordination, allowing us to establish a basic sensorimotor network for drawing in the brain, one that includes neural areas for emanation as central components but that are missing in all previous studies in which visual feedback was lacking.

## Materials and Methods

### Subjects

Fifteen right-handed subjects (9 females, mean age 25 years old, range 18–35 years old) participated in the study after giving their informed consent (McMaster Research Ethics Board, McMaster University). Handedness was tested using the Edinburgh handedness inventory [23]. Subjects had normal or corrected-to-normal vision and no history of neurological disorders, psychiatric illness, alcohol or substance abuse, and were not taking psychotropic medications. No subject required corrective lenses during the MRI experiment. Subjects received monetary compensation for their participation.

### Apparatus

Drawing was performed on an MRI-compatible (i.e., non-ferromagnetic) drawing tablet developed by Tam et al. [19], as connected to a Hewlett Packard Pavilion dv5 laptop computer running E-Prime 2.0 (Psychology Software Tools, Sharpsburg, PA). Figure 1 of Tam et al. [19] demonstrates the set-up of the tablet and its placement above a subject in an MRI scanner. The tablet consists of a resistive touch-screen connected to an elevated support platform. The tablet was custom-made to fit the specifications of the GE scanner-bed used in this study. A controller box served as an interface between the tablet and the laptop computer that was used for both stimulus presentation and the recording of drawing data. The dimensions of the screen were 12.8 cm width by 9.2 cm height. Drawing was made using a simple plastic stylus roughly the size and weight of a ballpoint pen. When subjects were placed in the scanner, the drawing tablet was fitted close to the body surface so as to permit easy access with the hands. The right hand was used for drawing (all subjects were right handed), and the left hand rested on the left side of the support platform. A series of calibration tasks was performed for each subject in order to ensure that the projected image was visible to them and that their drawings were well contained within the field of view of the LCD projector.

**Figure 1:**
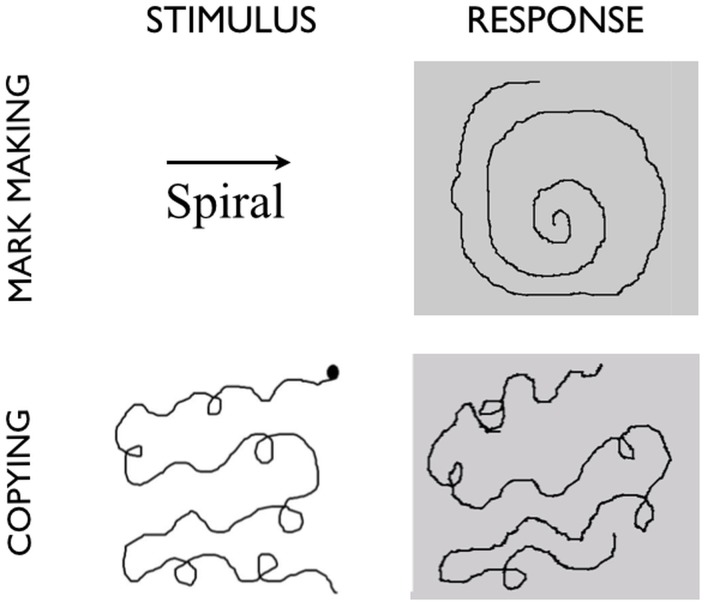
Tasks and stimuli for the study. Representative stimuli and responses for two of the drawing conditions. In mark making, the name of a geometric figure and a direction for drawing it are presented. Blind drawing (not shown in the figure) has exactly the same stimuli, but no response is observable on the drawing tablet. In copying, an object and a starting point for drawing are indicated, and the subject creates a copy of the object in the space to the right, with the object continually in view.

It is important to note that no drawings actually appeared on the tablet’s surface. All drawings were seen via a mirror positioned in the visor of the head coil. The LCD projector presented images onto this visor, and the light was reflected by the mirror to the subject’s eyes. This gave the veridical impression to subjects that their drawings were occurring on the tablet’s surface. However, this occurred indirectly through information from the computer screen projected onto the visor through the LCD projector. In addition, due to the arrangement of the tablet in the scanner bed, subjects were not able to see the drawing tool during drawing. Hence, the only dynamic visual stimulation that they received during drawing came from the emanating image and not from perceived movement of the stylus tip or their own hand.

### Stimuli

Two categories of stimuli were used in the production tasks (see Figure 1): 1) geometric patterns, of which there were three types (spirals, zigzags, and serpentines), and 2) embellished geometric patterns of the same three types (i.e., geometric patterns with added loops, used for the copying task).

### Tasks

Each participant took part in a one-hour training session on a day prior to the MRI scan in order to become proficient at using the drawing tablet while minimizing head movement as well as to practice the tasks to be performed in the scanner. Training was performed in a simulated scanner environment in which subjects were supine and had the tablet positioned across their lap. Subjects were positioned at a comfortable viewing position below a computer monitor that was mounted to a hinged arm on the wall. Pre-recorded scanner noise was played in the background as subjects performed each training task. During the scanning session, subjects performed each trial as an alternation between 20s periods of fixation and 20s periods of task. Each scan lasted 4 min and consisted of 6 trials of the same condition. During the fixation periods, a black fixation-cross was projected onto the center of a grey background. There were four scans altogether, one for each of the following conditions: perception, mark making, blind drawing, and copying. All stimuli were presented using E-Prime 2.0 running on a Hewlett Packard Pavilion dv5 laptop.

Participants performed the following three drawing tasks in random order. 1) *Mark making*: participants were prompted for 2s with the written name of a geometric pattern (zigzag, spiral, or serpentine) as well as an arrow indicating the direction in which to draw it (i.e., leftward or rightward). This occurred in the center of the screen. The prompt was then removed, and the subject drew the pattern from memory on the right half of the screen for the 18s remaining in the epoch. The subject was instructed to draw for the duration of the 18s epoch. If the subject reached the edge of the defined drawing space while drawing zigzags or serpentines, they were told to double back in the other direction until the task epoch was over. For all conditions, the drawing direction was balanced across stimuli. Subjects were unable to see either the stylus or their own hand. Thus, the only visual feedback available to them during the drawing tasks was the emanating image. 2) *Blind Drawing*: this was exactly the same as the mark making task except that the line color for drawing was changed to the background color of the display, thereby removing all visual feedback. This created a condition in which the subject could not see their drawing while making it, a situation common to many neuroimaging studies of drawing (see Introduction). 3) *Copying*: participants were presented on the left half of the screen with a visual model that was to be copied on the right half of the screen. The model remained visible throughout the task epoch. The stimuli were not the simple geometric stimuli used in the other mark making conditions but rather embellished geometric patterns in which loops were added to the geometric patterns (see Figure 1). The reason for this change was that pilot testing showed that using standard geometrics allowed subjects to ignore the features of the model and simply create the patterns from memory, just as they had in the mark making condition. The introduction of embellishments was a necessary step to keep the subject’s attention focused on the visual features of the model. For each stimulus, a starting point for copying was indicated on the model so as to balance drawing direction across stimuli. The full set of six copying stimuli is presented in Document S1. Finally, 4) a *Perception* task was performed in which subjects passively viewed animations of abstract line drawings unfurling on the projected screen over the course of 20s. Since pilot testing showed surprisingly widespread brain activations for this task, we had subjects perform it first so as to reduce any contamination of actual drawing on perception. The production tasks were then randomized among themselves after the perception task. For all drawing tasks, motor activity – and thus visual feedback – was limited to the right half of the tablet. Subjects were free to move their eyes during all conditions in order to make the drawing tasks naturalistic.

### Image Acquisition

Magnetic resonance images were acquired with a GE Medical Systems Signa Excite 3-Tesla MRI at the Imaging Research Centre at St. Joseph’s Healthcare Hamilton. The subject’s head was firmly secured in the head coil using foam pads placed around the ears. Ear plugs were used to help block out scanner noise.

Functional images sensitive to the blood-oxygen-level-dependent (BOLD) signal were collected with a gradient-echo echo planar imaging (EPI) pulse sequence using standard parameters (TR = 2000 ms, TE = 45 ms, flip angle = 90^o^, 31 slices, 4 mm slice thickness, no gap, matrix size = 64×64, field of view = 24 cm, voxel size = 3.75 mm×3.75 mm×4 mm), effectively covering the whole brain. All functional scans lasted 4 min, resulting in the collection of 120 brain volumes per scan.

High-resolution, T1-weighted structural images were acquired in order to register functional activity onto brain anatomy. The scanning parameters were 3D-FSPGR, IR-prepped, Ti = 450 ms, flip angle = 12 degrees, TR = 7.5 ms, TE = 2.1 ms, field of view = 240 mm×180 mm, slice thickness = 2 mm, acquisition matrix 320×192, 1 average (NEX = 1), receiver bandwidth = 31.25 kHz, data was interpolated to a 512×512 matrix, and the number of slices doubled during reconstruction, resulting in 164 slices.

### Data Analysis

Functional images were reconstructed offline, and the scan series was realigned and motion-corrected using BrainVoyager QX 2.4 (Brain Innovation, Maastricht). Motion-correction analysis revealed that subjects displayed very little head movement. Translational and rotational corrections did not exceed an acceptable level of 1.5 mm and 1.5 degrees, respectively, for any subject. During the preprocessing stage, a temporal high-pass filter was applied at a frequency of 0.0078 Hz, or 2 cycles per scan, using the GLM-Fourier algorithm. 3D spatial smoothing was performed using a Gaussian filter with a FWHM kernel size of 4 mm. Following realignment, each functional scan was normalized to the Talairach template [24]. The BOLD response for each task-block was modeled as the convolution of a 20s boxcar with a synthetic hemodynamic response function composed of two gamma functions. Beta weights associated with the modeled hemodynamic responses were computed to fit the observed BOLD-signal time course in each voxel for each subject using the General Linear Model, as implemented in BrainVoyager QX 2.4. The six head-motion parameters were included as nuisance regressors in the analysis. Each subject’s data was processed using a fixed-effects analysis, corrected for multiple comparisons using a Bonferroni correction at a threshold of p<0.05. Contrast images for each subject were brought forward into a random-effects analysis, where a false discovery rate (FDR) of p<0.01 was employed as a correction for multiple comparisons, with a cluster threshold of k = 25. Group data were registered onto the inflated brain of one of the subjects within the study (Subject 4), as generated using Brain Voyager. Talairach coordinates were extracted using NeuroElf (neuroelf.net).

## Results

**Figure 2:**
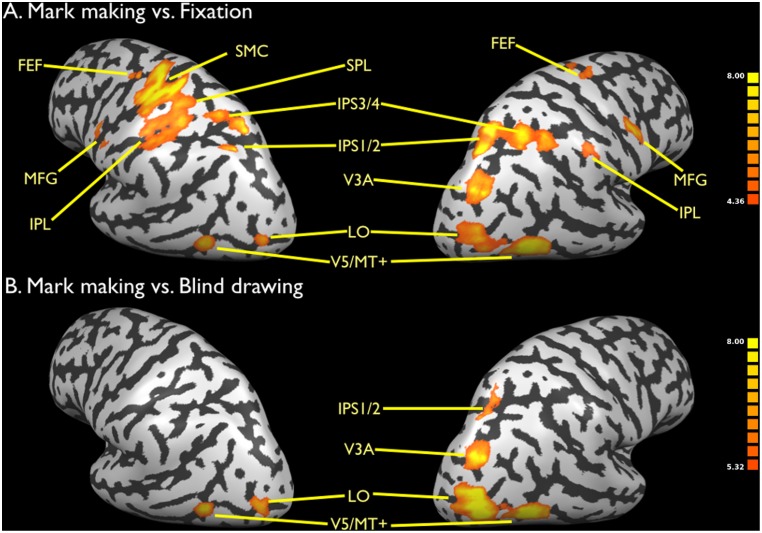
Brain activations for mark making. a) Mark making vs. fixation. b) Mark making vs. blind drawing. Data are corrected for multiple comparisons using FDR p<0.01. Activations shown in Figures 2–5 are rendered onto an inflated brain of one of the subjects in the study (Subject 4) as normalized into Talairach space. The color bars in Figures 2–5 reflect the t score of the activated voxels for a given contrast. Abbreviations: FEF: frontal eye fields; IPS1/2: segments 1 and 2 of the intraparietal sulcus; IPS3/4: segments 3 and 4 of the intraparietal sulcus; MFG: middle frontal gyrus; MT: middle temporal; SMC: sensorimotor cortex; SPL: superior parietal lobule.

Figure 1 provides examples of the stimuli used for the mark making and copying tasks, as well as representative drawn responses. Document S2 provides representative examples of drawn responses for the blind drawing condition. Figure 2a shows the activation pattern for mark making in contrast with fixation. The Talairach coordinates of the activations are listed in Table 1. Prominent activations related to motor control of the right hand and forearm were found in the left sensorimotor cortex and right posterior cerebellum (lobule V). Additional motor activations were found bilaterally in the frontal eye fields medial to the primary hand activations. Next, while no activation was found in the primary visual cortex, strong activations were found bilaterally in the motion-perception area V5/MT+ (BA 19) and in area LO posterior to it (BA 18). LO is a form-processing area but is thought to be important in processing form from motion [25]. Higher-level motion-related visual activations were seen dorsomedially in area V3A (BA 19) in the right hemisphere. As mentioned in the Methods sections, subjects in the scanner were unable to see the drawing tool, and hence all visual activations reported in this study result from perception of the emanating image, not perception of the moving tool, hence permitting a disambiguation of the two sources. No activity was detected in another well-studied motion-perception area, the posterior superior temporal sulcus (pSTS), which is more associated with the perception of body motion. Parietal activations were found in the inferior parietal lobule (IPL), IPS bilaterally, and in the superior parietal lobule (SPL) in the left hemisphere (BA 7), directly posterior to the sensorimotor cortex. The IPS activations included both the posterior regions referred to by Swisher et al. [26] as IPS1 and IPS2 and the anterior regions referred to as IPS3 and IPS4. Finally, bilateral activations were seen in the middle frontal gyrus (BA 6). In sum, mark making defined the basic motor-sensory components of the drawing network, reflecting the dynamic visual/hand and visual/eye coupling that occurs during the generation of marks and the emergence of images.

**Table 1:** Talairach coordinates for mark making, blind drawing, and the contrast between mark making and blind drawing.

	Mark making				Blind drawing				Mark making vs. Blind drawing			
	x	y	z	t	x	y	z	t	x	y	z	t
Right Hemisphere												
*Frontal Lobe*												
Middle frontal gyrus (BA6)	51	−1	37	12.4	63	2	46	7.3				
Precentral gyrus (BA 4)	27	−16	58	6.9								
Frontal eye fields (BA 6)	18	−13	58	6.7								
Inferior frontal gyrus (BA 6)					51	−1	28	6.6				
*Parietal Lobe*												
IPS1/2 (BA 7)	21	−67	52	11.8					27	−67	58	10.1
IPS1/2 (BA 7)									24	−58	46	7.4
IPS3/4 (BA 7)	27	−49	49	8.6	30	−43	46	6.3				
Inferior parietal lobule (BA 40)	51	−28	40	8.8								
Precuneus (BA 7)	12	−73	61	8.7								
Inferior parietal lobule (BA 40)	39	−40	43	8.4	51	−31	43	6.6				
*Occipital Lobe*												
V5/MT+ (BA19)	42	−67	−5	12.7					42	−67	−5	16.7
V3A (BA19)	30	−76	25	11.2					27	−76	25	9.6
LO (BA18)	33	−79	1	9.3					33	−76	−5	11.5
*Temporal Lobe*												
Middle temporal gyrus (BA 21/37)									45	−49	−5	9.2
*Cerebellum*												
Lobule V	24	−52	−14	8.2								
Left Hemisphere												
*Frontal Lobe*												
Sensorimotor cortex (BA 4/3)	−30	−28	67	12.7	−33	−28	67	9.4				
Middle frontal gyrus (BA 6)	−57	−1	37	7.7								
Frontal eye fields (BA 6)	−12	−16	55	7.7	−3	−16	58	7.5				
*Parietal Lobe*												
Somatosensory cortex (BA3)	−39	−28	49	12.0	−33	−28	49	8.5				
Postcentral gyrus (BA 2)					−36	−40	67	8.3				
IPS1/2 (BA7)	−21	−67	46	11.3								
Superior parietal lobule (BA 7)	−24	−67	55	8.2								
Paracentral lobule (BA 5)	−9	−31	49	7.7								
IPS3/4 (BA7)	−30	−49	52	7.6								
Inferior parietal lobule (BA 40)	−48	−34	40	7.6	−51	−37	46	5.0				
*Temporal Lobe*												
Fusiform gyrus (BA 37)									−39	−49	−11	6.3
*Occipital Lobe*												
LO (BA18)	−24	−82	13	8.8					−24	−82	13	11.1
V5/MT+ (BA19)	−42	−64	−2	8.8					−39	−64	−5	11.0
LO (BA18)	−30	−82	4	8.1								
Fusiform gyrus (BA 19)									−36	−79	−14	8.4
V3A (BA19)									−27	−79	31	6.9
*Cerebellum*												
Lobule V	24	−52	−14	8.2								
Lobule VI									−42	−55	−20	7.5

Stereotaxic coordinates are in millimeters along the left-right (x), anterior-posterior (y), and superior-inferior (z) axes. In parentheses after each brain region is the Brodmann area, except for the cerebellum, in which case the anatomical labels of Schmahmann et al. (2000) are used. Abbreviations: IPS: intraparietal sulcus; LO: lateral occipital complex; MT: middle temporal.

Turning off visual feedback while doing mark making created a condition of blind drawing, which places drawing under purely proprioceptive control and which serves as a motoric control for mark making. Figure 2b shows the contrast of mark making vs. blind drawing (the reverse contrast gave no signal). The Talairach coordinates of these activations and of those for blind drawing vs. fixation are present in Table 1. As expected, all of the motor activity in the left sensorimotor cortex and right cerebellum was eliminated in this subtraction due to the matched motoric nature of the tasks. What remained was the occipito-parietal visual-motion network, including areas V5/MT+, LO, V3A, and IPS1/2, with a strong right-dominant pattern. This group of areas represents the best neural correlate of the phenomenon of emanation occurring during drawing-based image generation.

**Figure 3:**
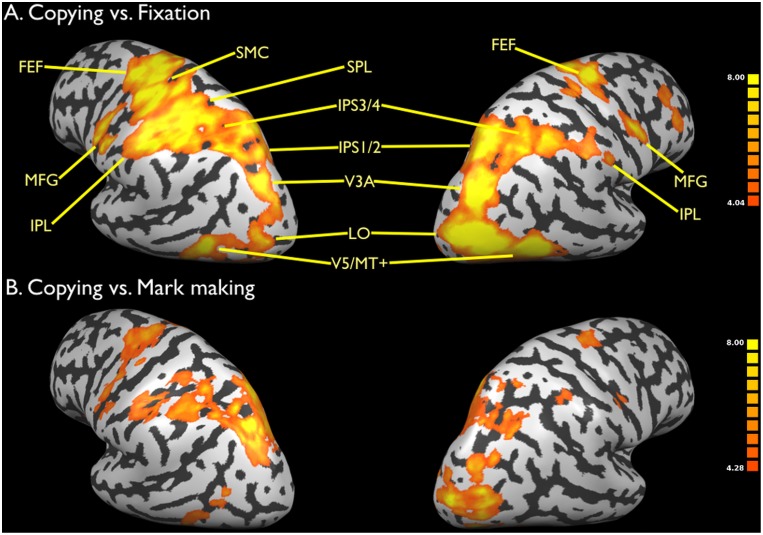
Brain activations for copying: Lateral view. a) Copying vs. fixation. b) Copying vs. mark marking. Data are corrected for multiple comparisons using FDR p<0.01. See legend to Figure 2 for abbreviations.

**Figure 4:**
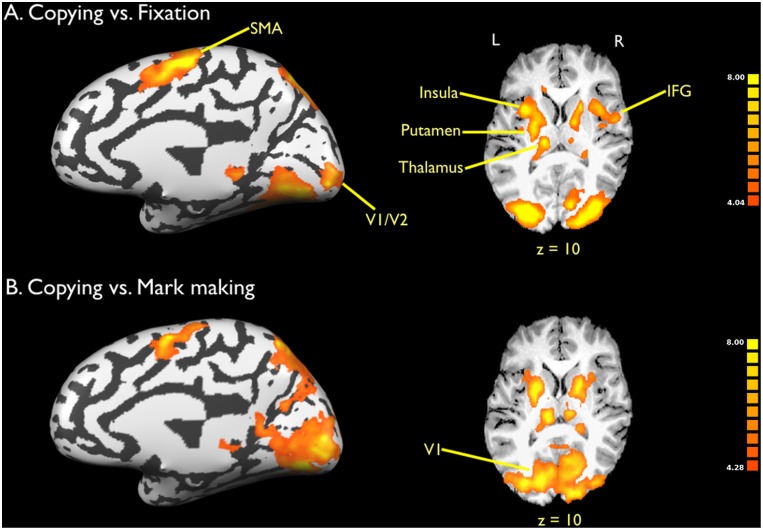
Brain activations for copying: Medial view. a) Copying vs. fixation. b) Copying vs. mark marking. Data are corrected for multiple comparisons using FDR p<0.01. The left side of the slices is the left side of the brain, as indicated by the L (left) and R (right) symbols. The Talairach z coordinate is shown below the slices. Abbreviations: IFG: inferior frontal gyrus; SMA: supplementary motor area.

Next, activations for copying versus fixation are shown in Figure 3a (lateral view) and Figure 4a (medial view). The Talairach coordinates for these activations are presented in Table 2. While copying showed activations in the same set of regions as mark making, the global level of activation for copying was much more intense than that for mark making, including in areas involved in emanation. In addition, two new systems were present for copying that were not seen in memory-based drawing, and this was highlighted in the contrast of copying vs. mark marking in Figures 3b and 4b. First, activations were seen in the basal ganglia system, including bilateral putamen and ventral thalamus. This system, perhaps in combination with the right IFG, most likely mediates the imitative aspect of copying. Second, strong activity was seen in the primary visual cortex and surrounding areas (BA 17 and 18). Such areas were not detected in mark making. This lower-level visual activity most likely reflects the presence of the static model that the subject had to glance at repeatedly during the drawing process. This result is consistent with the findings of Ferber et al. [18] who, in a similar contrast between copying and drawing from memory, found greater activity in the cuneus and other inferior occipital regions for copying.

**Table 2:** Talairach coordinates for copying and perception.

	Copying				Perception			
	x	y	z	t	x	y	z	t
Right Hemisphere								
*Frontal Lobe*								
SMA (BA 6)	3	−4	58	12.1				
Superior frontal gyrus (BA 6)					30	−4	67	9.4
Frontal eye fields (BA 6)	24	−13	52	11.7	24	−10	58	8.9
Middle frontal gyrus (BA 6)	54	2	40	9.8	45	−4	40	10.4
	36	−1	34	9.8				
	45	−1	28	8.1				
*Parietal Lobe*								
IPS1/2 (BA 7)	21	−64	46	11.9	21	−64	49	13.8
	30	−61	55	10.9				
IPS3/4 (BA 7)	33	−49	49	9.1	30	−46	46	11.6
Superior parietal lobule (BA 7)	18	−73	61	9.6	24	−70	61	10.0
Inferior parietal lobule (BA 40)	39	−37	40	10.4	39	−40	46	11.5
					54	−28	37	9.7
Somatosensory cortex (BA 1/2)					39	−37	73	8.6
Precuneus (BA 7)					6	−70	55	8.1
*Occipital Lobe*								
LO (BA 19/18)	24	−85	10	15.1	33	−79	7	10.5
	36	−79	1	14.9				
	39	−70	−5	10.6				
V3A (BA 19)	30	−70	22	13.7	30	−76	22	15.5
Lingual gyrus: V2/V1 (BA 18)	27	−82	1	12.7	15	−85	−2	9.4
	21	−73	1	11.8				
V5/MT+ (BA 19/37)	45	−58	−5	11.9	42	−64	−2	13.3
					45	−52	−5	11.5
Fusiform gyrus (BA 19)	33	−61	−11	10.4	24	−76	−11	13.2
*Subcortical*								
Pulvinar					21	−28	1	9.7
Caudate nucleus	21	14	13	9.6				
Putamen	15	−1	13	9.5				
Caudate nucleus	24	−7	25	8.5				
Globus pallidus	18	−7	4	8.0				
*Cerebellum*								
Lobule VI	6	−67	−14	8.2				
Left Hemisphere								
*Frontal Lobe*								
Sensorimotor cortex (BA 4/3)	−45	−16	58	13.9				
	−39	−28	52	10.9				
Sensorimotor cortex (BA 4)	−15	−22	73	9.0				
Frontal eye fields (BA 6)	−24	−16	52	12.3	−21	−13	55	13.4
Middle frontal gyrus (BA 6)	−45	−4	31	11.5				
SMA (BA6)	−6	−7	58	11.3				
	−9	−13	70	10.6				
*Parietal Lobe*								
Inferior parietal lobule (BA 40)	−39	−40	40	13.8	−39	−49	49	10.7
	−51	−31	37	11.4	−48	−37	43	8.1
	−24	−64	40	9.5				
IPS 3/4 (BA 7)	−33	−58	52	11.4				
	−33	−46	58	11.3				
Superior parietal lobule (BA 7)					−30	−67	64	13.0
					−33	−61	55	11.2
IPS1/2 (BA 7)	−24	−67	55	9.9	−24	−67	52	11.6
Postcentral gyrus (BA 3/1)	−33	−28	67	10.8				
Precuneus (BA 7)	−12	−73	52	10.1				
*Occipital Lobe*								
LO (BA 19)	−30	−79	13	14.9	−30	−79	13	10.9
V5/MT+ (BA 19)	−39	−67	1	12.5	−39	−64	−8	10.2
V3A (BA 19)	−30	−76	22	11.2				
Lingual gyrus: V1/V2 (BA 17/18)	−12	−88	−8	9.1	−12	−88	−5	8.1
Inferior occipital gyrus (BA 18)	−33	−85	−5	9.1				
Lingual gyrus: V1 (BA 17/18)	0	−85	−5	8.5				
Fusiform gyrus (BA 19)					−30	−79	−11	10.4
*Subcortical*								
Caudate body	−21	−7	22	13.4				
Claustrum	−27	11	13	12.4				
Thalamus	−12	−16	13	9.5				
Pulvinar	−3	−31	4	9.0	−6	−28	1	9.8
	−24	−28	4	8.5	−24	−28	4	9.4
*Cerebellum*								
Lobule VI	−27	−61	−14	9.5				

Stereotaxic coordinates are in millimeters along the left-right (x), anterior-posterior (y), and superior-inferior (z) axes. In parentheses after each brain region is the Brodmann area, except for the cerebellum, in which case the anatomical labels of Schmahmann et al. [46] are used. Due to the excessive number of activation foci for copying and perception, we decided to eliminate foci with a t value less than 8.0. The data in Table 1, by contrast, includes activation foci with a t value as low as 6.3. Abbreviations: IPS: intraparietal sulcus; LO: lateral occipital complex; MT: middle temporal; SMA: supplementary motor area.

**Figure 5:**
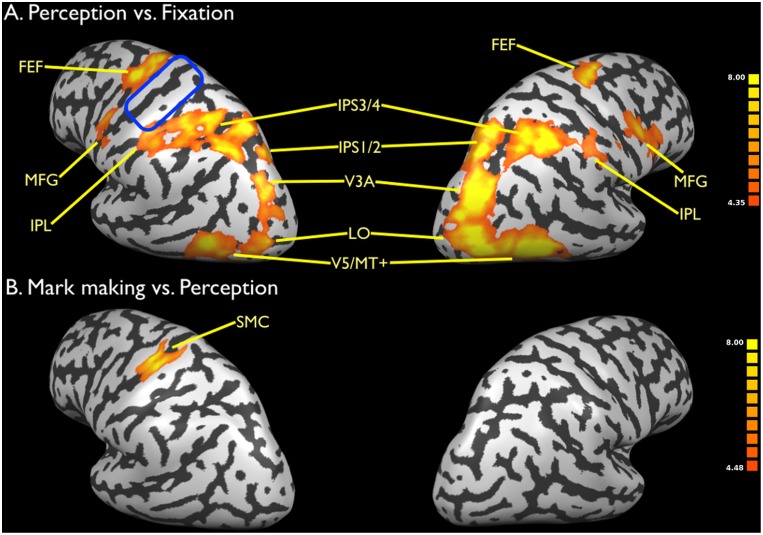
Brain activations for motion perception. a) Perception vs. fixation. b) Mark making vs. perception. Data are corrected for multiple comparisons using FDR p<0.01. The blue oval in panel a indicates the region of the sensorimotor cortex for copying not activated in motion perception. See legend to Figure 2 for abbreviations.

Finally, we examined drawing perception alone by showing subjects animations of emanating images of abstract patterns (Figure 5a). The Talairach coordinates of these activations are presented in Table 2. Since pilot testing had revealed that this condition gave very strong activity throughout the drawing network, we decided to place this condition first in the scanning session in order to reduce any carryover effects that might come from performing drawing itself. As with the pilot data, the group results showed very strong activity throughout the drawing network, with the exception of the primary motor cortex. Brain areas associated with emanation were strongly activated in this condition, again with a right-hemisphere dominance, just as in production. Figure 5b shows the subtraction of mark making vs. perception. As can be seen, this subtraction eliminated virtually all of the activations for mark marking (compared with Figure 2a), except for the primary motor cortex (Talairach coordinates −36, −25, 55) and contralateral cerebellum (Talairach coordinates 15, −52, −14). Another way of thinking about this subtraction is that it basically resulted in the brain activity produced by blind drawing (see Table 1).

## Discussion

We used functional MRI to explore the neural basis of the generation of images through mark making, including its defining property of emanation. Our use of an MRI-compatible drawing tablet allowed us to manipulate visual feedback, in contrast to many previous studies of drawing, where subjects obtained no feedback of their drawing activity. The blind drawing task essentially mimicked the situation of all overt drawing studies in which subjects did not have access to visual feedback (i.e., through drawing on a pad or drawing in the air) and hence did not perceive image formation. Providing visual feedback to subjects using the projected display created the more naturalistic situation of subjects perceiving images as they generated them in real time, hence allowing us to identify components of the motion-perception system of the brain’s dorsal visual stream that mediate the perception of the emanating image.

The process of mark making from memory, involving the production of uninterrupted geometric patterns, defined the basic components of the drawing network, with brain areas involved in hand movement (M1, SMA, cerebellum), eye movement (FEF), visual motion perception (V5/MT+, V3A, LO), and sensorimotor coupling (IPS, IPL, SPL). Activity in this system as a whole was modulated quantitatively by the allocentric requirements of the drawing task, being lowest for blind drawing and highest for copying and perception. Emanation was associated with an occipito-parietal stream along the posterior aspect of the brain, extending dorsomedially from V5/MT+ to the posterior IPS, and encompassing the motion-related area V3A. To our surprise, passive perception of emanation was an extremely strong stimulus for the drawing network, eliciting activity in the areas involved in motor planning, even though subjects were explicitly instructed to passively view the emanation presented to them. This finding might suggest that some motor planning processes are automatic and are not under conscious control. In contrast, blind drawing gave only the motor components of the system plus activity in the left SPL.

### Emanation: Pictures Result from Trailing

It is interesting to note that the neural system for drawing is strikingly similar to that for gesture production [27]. We can classify drawing movements as a form of instrumental (transitive) gesture. From a cognitive standpoint, drawing might simply be gesturing that leaves a trail behind. Studies of drawing in which subjects moved their finger in the air ([4]; see also [28]–[30] for writing) are, in reality, studies of pantomime production. Ekman and Friesen [31] referred to gestures of this type as “pictographs”, making an allusion to drawing. Given the longstanding interest in the gestural origins of language through processes like pantomime [32]–[33], it might be the case that figurative drawing emerged from iconic gesturing processes like pantomime through the realization that such movements could leave a trail behind, perhaps first occurring using fingers or sticks in media like the earth or sand or even on the human body. In this regard, a key area that mediates emanation, namely V3A, appears to have undergone evolutionary modification in humans compared to monkeys [34]. This neural change might have relevance to the evolution of species-specific capacity for drawing in humans.

An important visual component of drawing compared to most other visuomotor tasks that people engage in is that visual information *accumulates* through trailing as the drawing progresses. The comparison between mark making and blind drawing revealed an occipito-parietal stream extending up the posterior aspect of the brain from V5/MT+ through V3A to the posterior IPS, with right hemisphere dominance during both production and perception. This stream was also active during copying and the passive perception of emanating images. Since subjects could not see the drawing tool in our experimental set-up, visual emanation could only come from the image alone and not from perception of the hand or drawing tip, thereby disambiguating these various sources of visual stimulation. Activity in this occipito-parietal stream is thus a neural marker of emanation, as shown in other studies of drawing in which visual feedback was present during image generation due to the use of MRI-compatible devices. In particular, our results are concordant with the contrast between visual feedback and no visual feedback in Thaler and Goodale’s [20] analysis of line drawing.

Another brain area important for high-level motion perception, namely the pSTS [35]–[36], was not active in any of the conditions in this study. This is in distinction to many studies of gesture perception, where the pSTS is commonly seen [37]–[38]. This supports the association of the pSTS with the perception of biological motion, namely the motion of articulated bodies that move in the manner typical of animals [39]. An ALE meta-analysis of action observation and imitation [27] reported activity in V5/MT+, pSTS and IPS, but not in V3A. These findings suggest that the emanation system is engaged more strongly by trailing than by the perception of hand or body movement alone. The preliminary conclusion from this is that the pSTS, but not V3A, is activated by the perception of others’ gestures and actions, and that V3A, but not the pSTS, is activated by the perception of emanation during drawing and writing.

Why might V3A be a critical area for drawing emanation when it seems not to respond to the perception of biological motion? V3A is well known to be directionally-selective and to be responsive to coherent motion compared to random motion [40]. It might therefore be involved in extracting form from motion [41], a function that is of importance in drawing, since form unfolds over time through a motion-based process of trailing. For example, Ellamil et al. [17], in a study of creative drawing using the same drawing tablet employed in this study, found right V3A to be active during the generative phase of drawing compared to an evaluative phase that followed it. In addition, V3A has also been shown to be responsive to ego-motion, in other words self-motion through space [40]–[41]. Thus, in contrast to the pSTS’s responsiveness to the motion of others, V3A, along with areas like V6 and the IPS [42], might be more responsive to the motion of oneself. The optic flow that is perceived during emanation in our experiment is paradoxical in that it is based neither on an object moving through space nor on the subject moving through space relative to a fixed spatial reference frame. Instead, it represents the *outcome* of self-generated movement and is thus a proxy for self-motion. Although our MRI set-up dissociated emanation from hand and tool movement, there is a strong correlation between the motions of the hand, drawing tool and the emerging image during naturalistic drawing. So, V3A activation in our experiment might represent a response to neither object motion nor self-motion per se but instead to self-generated motion. It is expected that the emanation system would be even more engaged if the hand and drawing tool were perceivable during drawing. Although subjects were not able to see their hand or the drawing tool in our set-up, it would be quite interesting to compare the effects of viewing hand or drawing-tool movement without emanation vs. the emanation without perception of hand/tool movement that occurred in our set-up. Finally, the V3A activations in our study were adjacent to a region called SPOC (superior parieto-occipital cortex [43]) that is implicated into visuomotor functions related to reaching, pointing, and grasping. This area seems to be involved in encoding motor affordances, such as the reachability of an object by the hand. This might have relevance not only to our tool-based drawing tasks but to our motion-perception condition as well, especially if this task was processed by subjects as a type of virtual drawing.

### Limitations

Two additional issues that would be important to explore in order to develop a more comprehensive understanding of the drawing system of the brain are figurativity and flattening. The current study used geometric figures as stimuli, but it is important to look at more-quotidian items as drawing stimuli as well, such as cars or houses. Harrington, Farias and Davis [11] performed a comparison between the copying of figurative vs. abstract models, but did so using visual imagery in the absence of actual drawing. Their results demonstrated overall similarity between these two categories of stimuli but significant differences in the fusiform gyrus, basal ganglia, and inferior frontal gyrus. The fusiform gyrus is part of the visual ventral stream, and so its preferential activation for figurative compared with abstract images might be indicative of the “object” status of figurative items in the object-recognition pathway of the inferior temporal lobe.

Finally, copy-based drawing of natural scenes requires a dimensional reduction (i.e., a flattening) from the three dimensions of visual perception to the two dimensions of the drawing space. Artists work extensively with monocular depth cues in order to create a sense of three-dimensional perspective in drawn images [44]. How they achieve this is not understood at the neural level. What is better understood is the perceptual system involved in depth processing [45]. Georgieva et al. [34] carried out a study of depth processing based on binocular-disparity cues and found an activation profile in motion-perception areas very similar to our occipito-parietal stream, including areas V5/MT+, V3A, V7/VIPS, and the posterior IPS. This implies an overlap in this system between disparity-based depth perception and the perception of trailing. This system might therefore be engaged in artists not only when they perceive depth in a model to be drawn but also when they transform its three-dimensional features into a two-dimensional form during the process of image generation.

## Supporting Information

Document S1 (PDF)

Document S2 (PDF)
