# Supplementary material for: The Neural Basis of Mark Making: A Functional MRI Study of Drawing
Source: PLoS One. 2014 Oct 1;9(10):e108628. doi: 10.1371/journal.pone.0108628 (PMC4182721; doi:10.1371/journal.pone.0108628)
Supplement: Document S1 — (PDF) [file pone.0108628.s001.pdf]

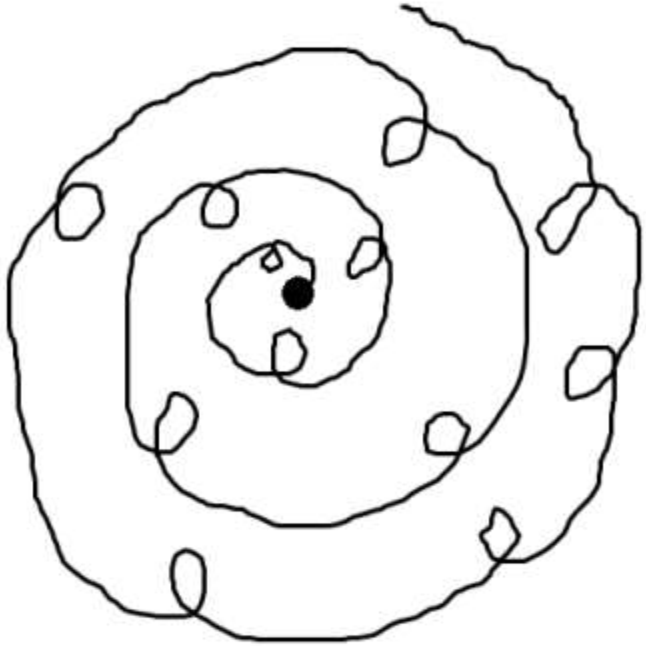

Counterclockwise Spiral

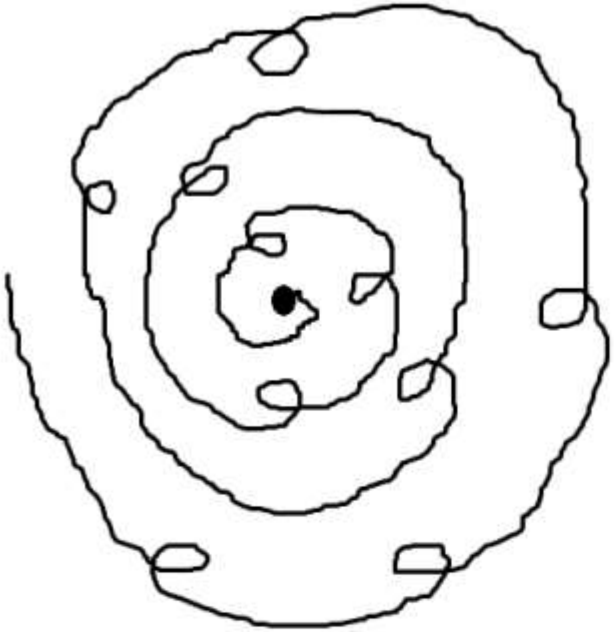

Clockwise Spiral

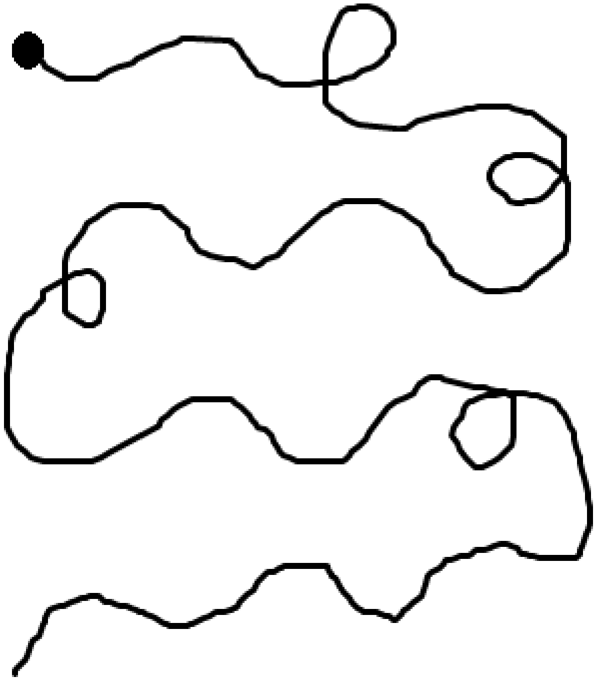

Left-to-right serpentine

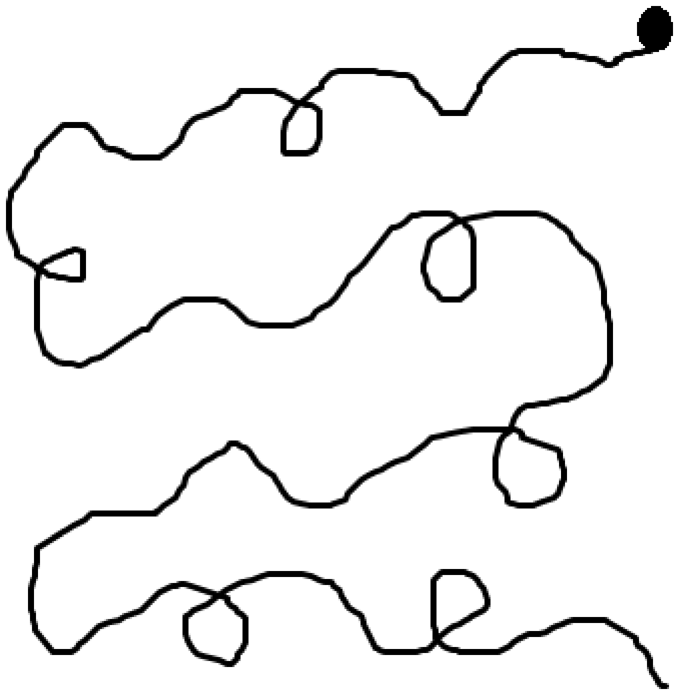

Right-to-left serpentine

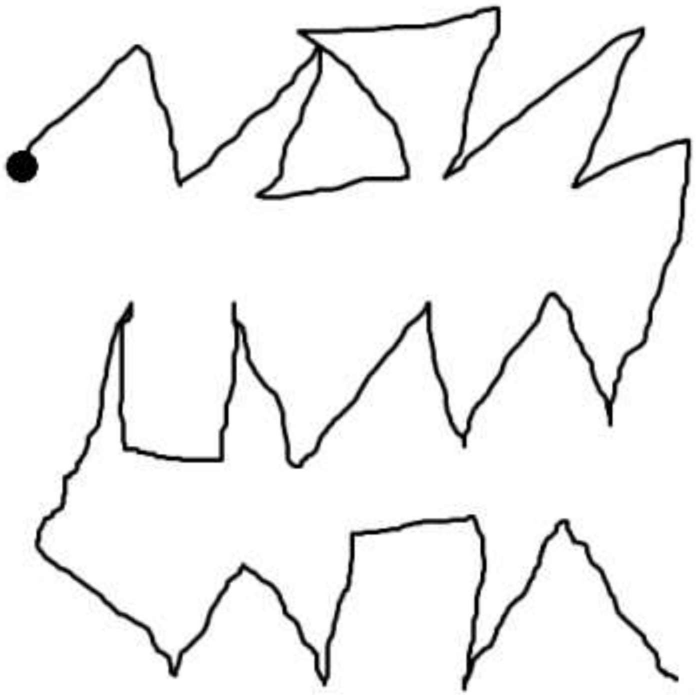

Left-to-right zigzag

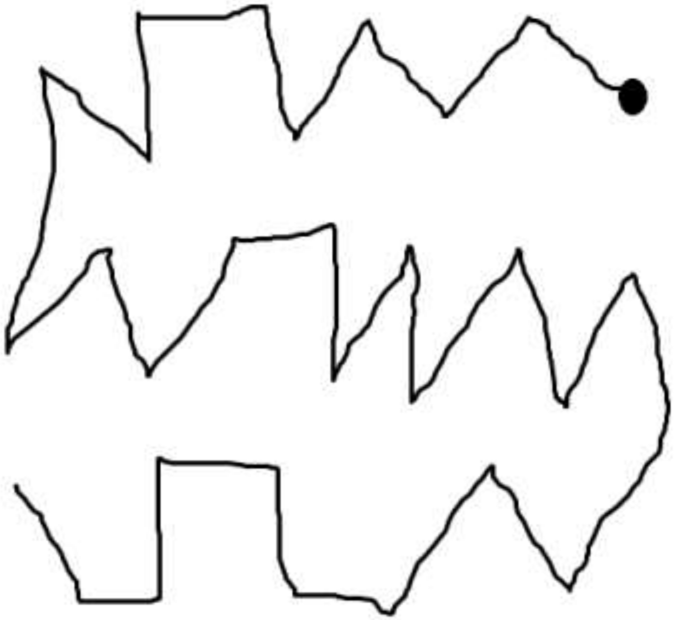

Right-to-left zigzag
